# Beliefs about medicines in relation to the initiation of cardiovascular preventive medications during a 3 year follow-up period after inclusion in the VIPVIZA trial: a cohort study

**DOI:** 10.1136/bmjopen-2025-100924

**Published:** 2025-12-23

**Authors:** Eva Sönnerstam, Henrik Holmberg, Bo Carlberg, Margareta Norberg, Anders Själander, E-L Glader

**Affiliations:** 1 Department of Medical and Translational Biology, Umeå University, Umeå, Sweden; 2 Department of Epidemiology and Global Health, Umeå University, Umeå, Sweden; 3 Department of Public Health and Clinical Medicine, Umeå University, Umeå, Sweden

**Keywords:** Cardiovascular Disease, Drug Therapy, Medication Adherence, Primary Prevention

## Abstract

**Objectives:**

To investigate if beliefs about medicines affect the time to the initiation of cardiovascular preventive medications during a 3 year follow-up period.

**Design:**

A questionnaire and register-based cohort study.

**Setting:**

Primary care in Sweden, in which people 40, 50 and 60 years old underwent risk factor screening and individual health promotion within the Västerbotten intervention programme (VIP).

**Participants:**

People at low/medium risk of cardiovascular disease (CVD) according to the risk factor screening were included in the **VI**suali**Z**ation of asymptomatic **A**therosclerotic disease for optimum cardiovascular prevention—a population-based Pragmatic Randomised Open Blinded End-point trial (PROBE) nested in the **V**ästerbotten **I**ntervention **P**rogramme (VIPVIZA), aiming at improved primary prevention of CVD. People participating in the VIPVIZA 3 year follow-up (n=3167 (89.7%)), receiving the Beliefs about medicines questionnaire (BMQ) (n=2314 (73.1%)) and with complete answers to at least one subscale in the BMQ general (n=2258 (97.6%)) were included. Moreover, only those 60 years old at baseline (n=2073 (58.7%)) and without antihypertensive and/or lipid-lowering drugs (n=1769 (50.1%)) 6 months prior to inclusion in the VIPVIZA trial were included. Accordingly, the final study population comprised 888 people without antihypertensive medicines and 1185 without lipid-lowering drugs, respectively.

**Measures:**

The primary outcome was time to the binary event of initiating antihypertensives or lipid-lowering agents, identified within the time frame from inclusion in the VIPVIZA study until the study participants’ 3 year follow-up visit. General beliefs about medicines were assessed according to the BMQ. Cox proportional hazards models, adjusted for sex, were conducted to investigate primary outcome.

**Results:**

Participants with stronger general beliefs about medicines being overused had significantly longer time to initiation of antihypertensive drugs in the control group (HR 0.91; 95% CI 0.84 to 0.996) but not in the intervention group (HR 1.05; 95% CI 0.95 to 1.16). No significant associations were found between beliefs about medicines and initiation of lipid-lowering treatment.

**Conclusions:**

A more negative perception of drugs being overused was significantly associated with delayed initiation of antihypertensive drug treatment. Our results suggest that the VIPVIZA intervention may overbridge negative perceptions and affect the initiation of antihypertensive medications in a positive manner.

**Trial registration number:**

NCT01849575 (date of registration: 8 May 2013).

**STRENGTHS AND LIMITATIONS OF THIS STUDY**

- The pragmatic study setting reflects the clinical reality among people with low and medium risk of cardiovascular disease.
- Beliefs about medicines were assessed using the previously utilised and validated Beliefs about medicines questionnaire (BMQ).
- The BMQ was consecutively distributed among a subsample of participants at 3 year follow-up, and the response rate was high.
- Baseline characteristics did not differ significantly between people who received the BMQ and those who did not, nor between people with full response and those with at least one missing on the BMQ.
- It is not possible to compare the participants’ beliefs about medicines at 3 year follow-up with the BMQ result at baseline since the BMQ questionnaire was only distributed at the 3 year follow-up.

## Introduction

 Atherosclerotic cardiovascular disease (CVD) is a major contributor to global morbidity as well as mortality.^1^ Among all CVD events, 60–70% are presented among people considered being at low or moderate risk for CVD, categorised according to the risk assessment model Systemic Coronary Risk Estimation (SCORE)^2^ and updated SCORE 2.^3^ For prevention, there is strong evidence for a healthy lifestyle and treatment with medications such as antihypertensive and lipid-lowering drugs.^3^ The treatment decision should be based on SCORE/SCORE 2 as well as the total cardiovascular risk.^2 3^ Cardiovascular preventive medications are effective and well proven but still underused.^3^,^6^

In the randomised controlled trial **VI**suali**Z**ation of asymptomatic **A**therosclerotic disease for optimum cardiovascular prevention—a population-based Pragmatic Randomised Open Blinded End-point trial (PROBE) nested in the **V**ästerbotten **I**ntervention **P**rogramme (VIPVIZA), the intervention was shown to lower the cardiovascular risk compared with the control group.^7 8^ In the VIPVIZA study, the intervention also increased the willingness to prescribe lipid-lowering drugs.^9^ Moreover, the robustness of the intervention effect was shown in another study where the prescription and initiation of lipid-lowering drugs increased after the 3 year follow-up when also the control group received the intervention.^10^ This highlights that besides the efficacy of the intervention on the CVD risk previously reported,^7 8^ the prescription of medication by the physician and the use of medication by the patient defines an important part of the intervention effect.

The perception of the expected benefits and risks of the medication predicts the willingness to receive a prescription and to use the drug.^11^ It has been reported that general beliefs of medicines being harmful and overused negatively affect the use of cardiovascular preventive medications.^12^ Among stroke survivors, no association was found between the beliefs about medicines and primary non-adherence to preventive drugs.^13^

Irrespective of changes in health status, general beliefs about medicines have been shown to be stable over time.^14^ However, an intervention with telephone counselling made by pharmacists showed that the perception of risks and benefits about medicines can, although moderately, be affected when measured by the Beliefs about medicines questionnaire (BMQ).^15^

The BMQ is a validated and well-established instrument.^16^ It was developed to distinguish between general beliefs and specific (subject’s own drug treatment) beliefs about medicines and can be used to compare specific beliefs between patient groups and general beliefs between populations including healthy persons.

The association between the patients’ beliefs about medicines and the use of preventive cardiovascular drugs has been more extensively studied in relation to drug adherence in terms of implementation, but the association with drug initiation, that is, the first step in the adherence process, is less explored.^12^ Moreover, whether patients’ beliefs about medicines affect how fast they initiate recommended CVD-preventive medications is unknown. Since the effect of drug treatment is dependent on adherence,^17^ it is important to gain further knowledge about this process to further improve cardiovascular preventive strategies. Accordingly, the aim with this VIPVIZA-based study was to investigate if the participant’s beliefs about medicines affect the time to initiation of antihypertensive and lipid-lowering medications during a 3 year follow-up period after inclusion in the VIPVIZA trial.

## Methods

### Study population and definitions

The participants in the present questionnaire and register-based cohort study, described according to Strengthening the Reporting of Observational Studies in Epidemiology (STROBE) cohort reporting guidelines,^18^ participated in the Pragmatic Randomised Open Blinded End-point (PROBE) VIPVIZA trial, which included mainly people at low/medium risk of CVD.^7^ The study protocol is presented in online supplemental appendix 1. Individuals who underwent risk factor screening and individual health promotion within the Västerbotten intervention programme (VIP) at age 40, 50 or 60 years and fulfilled one of the criteria below were in April 2013 to June 2016 also invited to the VIPVIZA trial. The inclusion criteria in the VIPVIZA study were:

1. Age 40 years with a first degree relative with a history of CVD before 60 years of age.
2. Age 50 years and at least one designated risk factor (smoking, diabetes, hypertension, serum low-density lipoprotein cholesterol level ≥174 mg/dL (4.5 mmol/L), abdominal obesity, a first degree relative with a history of CVD before 60 years of age).
3. Age 60 years.

#### Intervention and control

A randomisation list, generated by a computer before inclusion, was used to randomly assign participants 1:1 to either the intervention or the control group. The baseline VIPVIZA intervention consisted of pictorial information and motivational follow-up with a nurse based on a carotid ultrasound examination.^8 19^ In the intervention group, the pictorial information was given to both the individual and the general practitioner at the healthcare centre. The information is presented in online supplemental appendix 2 and comprised a simplified picture of the carotid arteries as well as the presence (red dot) or absence (green dot) of plaque and the participant’s estimated vascular age in relation to participants with the same sex and age in a reference population,^20^ based on the carotid intima media thickness (IMT). Carotid plaque was defined according to the Mannheim consensus.^19 21^ Vascular age was expressed with a gauge ranging from 10 years younger to 10 years older as colour codes from green over yellow and orange to red. Written information was also given about atherosclerosis being a dynamic process that potentially can be reduced, or even reversed, through healthier lifestyle habits and, if indicated, preventive pharmacological treatment.^2 22^ Detailed information about the pictorial and written information was previously given.^23^ A nurse made a phone call to the participant 2–4 weeks after carotid examination to make sure that the patient had received and understood the information. Furthermore, a dialogue following the methodology of Motivational Interviewing promoting healthy lifestyles and preventive medication (if needed) was held.^24^ Along with the identical pictorial and written information to the physician, information about how to interpret this information was also provided within the intervention, highlighting that the presence of plaque indicates very high risk of CVD.^2^ This information was not given to the participants in the control group, that is, the control group performed the ultrasound carotid examination, but the patients and their physicians did not receive any information about the results. After 1 year, risk factors were re-evaluated among participants in both the control and intervention group, and the result from this follow-up was provided to all study participants and their physicians. All decisions regarding preventive strategies were taken within primary care without any interference from the study team. People with severe carotid stenosis according to the vascular ultrasound at baseline were excluded from the study and referred to specialised care directly (n=22 out of 4177 screened). A more detailed description is given in previous publications.^8 23^

#### Data collection

For both groups, information on risk factors was collected from the VIP examination at baseline.^7^ The 3 year follow-up examinations were performed between September 2016 and May 2019.^8^ The mortality rate was only 13 participants in the intervention group and seven participants in the control group, corresponding to 0.6% of the total study population.^8^ In March 2017, the BMQ was added to the VIPVIZA questionnaire.^16^ Thus, from that timepoint, BMQ was given to all participants and only at the 3 year follow-up visit. Accordingly, there was no selection of participants receiving the BMQ questionnaire and baseline characteristics did not differ significantly, neither between people who received the questionnaire and those who did not, nor between those with full response and those with at least one missing on the BMQ (data not shown).

#### Inclusion criteria

**Figure 1:**
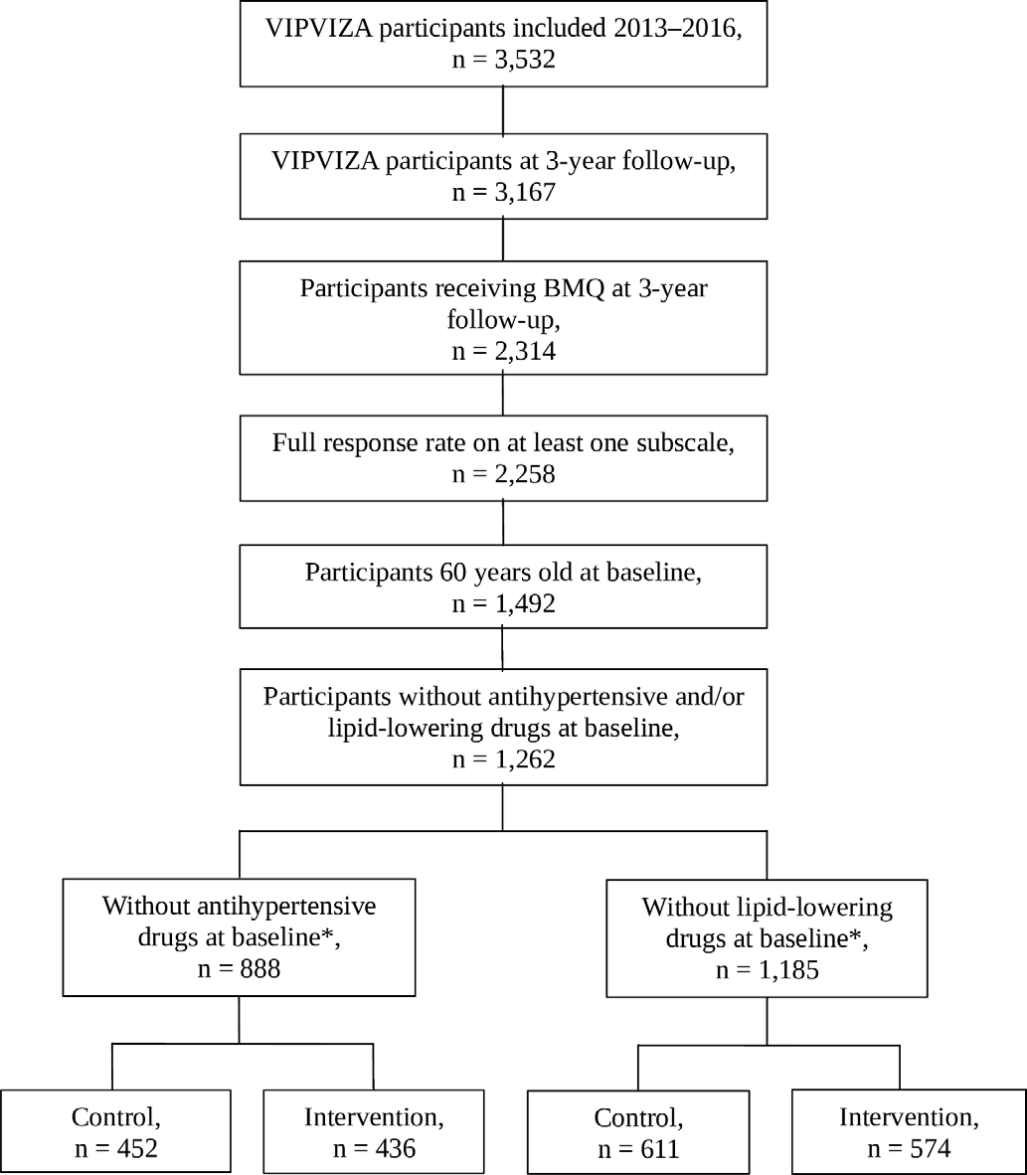
Flow chart over study population. *The participants without antihypertensive drugs and participants without lipid-lowering drugs are partly overlapping. BMQ, Beliefs about medicines questionnaire; VIPVIZA, **VI**suali**Z**ation of asymptomatic **A**therosclerotic disease for optimum cardiovascular prevention—a population-based Pragmatic Randomised Open Blinded End-point trial (PROBE) nested in the **V**ästerbotten **I**ntervention **P**rogramme.

To avoid confounding due to different inclusion criteria by age, only participants from the control and intervention groups who were 60 years old were included in the current study. Moreover, only those who received the BMQ questionnaire and with complete answers on at least one subscale, that is, answering all four questions on the subscales *overuse, harm* and *benefit,* respectively, were included in the present study on beliefs about medicines and initiation of cardiovascular preventive medications defined as antihypertensive (Anatomical Therapeutic Chemical (ATC) C03A, C07-09) and lipid-lowering drugs (ATC C10). Participants without dispensed lipid-lowering or antihypertensive agents, respectively, 6 months prior to inclusion in the VIPVIZA study were included in the analyses. A flow chart over the study population in the present study is shown in figure 1. Basic characteristics are presented in table 1.

**Table 1:** Baseline characteristics of the study population

	People without antihypertensive drugs			People without lipid-lowering drugs		
	Control (n=452)	Intervention (n=436)	Overall (n=888)	Control (n=611)	Intervention (n=574)	Overall (n=1185)
Sex						
Men	173 (38.3%)	191 (43.8%)	364 (41.0%)	249 (40.8%)	264 (46.0%)	513 (43.3%)
Women	279 (61.7%)	245 (56.2%)	524 (59.0%)	362 (59.2%)	310 (54.0%)	672 (56.7%)
Ultrasound year						
2013	0 (0%)	1 (0.2%)	1 (0.1%)	0 (0%)	1 (0.2%)	1 (0.1%)
2014	110 (24.3%)	102 (23.4%)	212 (23.9%)	153 (25.0%)	138 (24.0%)	291 (24.6%)
2015	189 (41.8%)	189 (43.3%)	378 (42.6%)	244 (39.9%)	236 (41.1%)	480 (40.5%)
2016	153 (33.8%)	144 (33.0%)	297 (33.4%)	214 (35.0%)	199 (34.7%)	413 (34.9%)
Body mass index						
Mean (SD)	26.3 (4.49)	26.2 (3.96)	26.3 (4.24)	26.9 (4.51)	26.8 (4.23)	26.8 (4.38)
Missing	–	–	–	1 (0.2%)	–	1 (0.1%)
Waist circumference						
Mean (SD)	92.9 (12.4)	93.1 (11.0)	93.0 (11.7)	94.4 (12.6)	94.8 (11.6)	94.6 (12.1)
Missing	8 (1.8%)	5 (1.1%)	13 (1.5%)	11 (1.8%)	9 (1.6%)	20 (1.7%)
Systolic blood pressure (mm Hg)						
Mean (SD)	127 (14.6)	127 (14.6)	127 (14.6)	130 (15.3)	131 (16.7)	130 (16.0)
Diastolic blood pressure (mm Hg)						
Mean (SD)	80.3 (9.04)	80.3 (8.82)	80.3 (8.93)	82.2 (9.43)	83.0 (10.1)	82.6 (9.75)
Missing	1 (0.2%)	0 (0%)	1 (0.1%)	1 (0.2%)	–	1 (0.1%)
LDL (mmol/L)						
Mean (SD)	3.73 (0.94)	3.77 (0.95)	3.75 (0.94)	3.70 (0.87)	3.70 (0.85)	3.70 (0.86)
Missing	3 (0.7%)	3 (0.7%)	6 (0.7%)	3 (0.5%)	2 (0.3%)	5 (0.4%)
HDL (mmol/L)						
Mean (SD)	1.53 (0.48)	1.51 (0.45)	1.52 (0.46)	1.50 (0.46)	1.47 (0.43)	1.48 (0.44)
Total cholesterol (mmol/L)						
Mean (SD)	5.87 (1.02)	5.88 (1.03)	5.87 (1.03)	5.81 (0.93)	5.79 (0.91)	5.80 (0.92)
Presence of plaque						
No plaque	219 (48.5%)	227 (52.1%)	446 (50.2%)	294 (48.1%)	284 (49.5%)	578 (48.8%)
Plaque	233 (51.5%)	207 (47.5%)	440 (49.5%)	317 (51.9%)	288 (50.2%)	605 (51.1%)
Missing	–	2 (0.5%)	2 (0.2%)	–	2 (0.3%)	2 (0.2%)
Score						
Low risk (<1%)	211 (46.7%)	184 (42.2%)	395 (44.5%)	259 (42.4%)	215 (37.5%)	424 (40.0%)
Moderate risk (1–4%)	231 (51.1%)	246 (56.4%)	477 (53.7%)	337 (55.2%)	339 (59.1%)	676 (57.0%)
High risk (5–9%)	9 (2.0%)	6 (1.4%)	15 (1.7%)	14 (2.3%)	18 (3.1%)	32 (2.7%)
Very high risk (≥10%)	0 (0%)	0 (0%)	0 (0%)	0 (0%)	0 (0%)	0 (0%)
Missing	1 (0.2%)	–	1 (0.1%)	1 (0.2%)	2 (0.3%)	3 (0.3%)
Framingham						
Low risk (<5%)	64 (14.2%)	50 (11.5%)	114 (12.8%)	61 (10.0%)	49 (8.5%)	110 (9.3%)
Light risk (5–9%)	162 (35.8%)	161 (36.9%)	323 (36.4%)	186 (30.4%)	177 (30.8%)	363 (30.6%)
Moderate risk (10–19%)	165 (36.5%)	157 (36.0%)	322 (36.3%)	231 (37.8%)	214 (37.3%)	445 (37.6%)
High risk (20–39%)	55 (12.2%)	63 (14.4%)	118 (13.3%)	126 (20.6%)	115 (20.0%)	241 (20.3%)
Very high risk (≥40%)	3 (0.7%)	4 (0.9%)	7 (0.8%)	4 (0.7%)	16 (2.8%)	20 (1.7%)
Missing	3 (0.7%)	1 (0.2%)	4 (0.5%)	3 (0.5%)	3 (0.5%)	6 (0.5%)
VIPVIZA vascular age						
Green	54 (11.9%)	42 (9.6%)	96 (10.8%)	76 (12.4%)	61 (10.6%)	137 (11.6%)
Yellow	83 (18.4%)	104 (23.9%)	187 (21.1%)	119 (19.5%)	127 (22.1%)	246 (20.8%)
Orange	124 (27.4%)	123 (28.2%)	247 (27.8%)	165 (27.0%)	152 (26.5%)	317 (26.8%)
Red	191 (42.3%)	167 (38.3%)	358 (40.3%)	251 (41.1%)	234 (40.8%)	485 (40.9%)

BMQ, beliefs about medicines questionnaire; SCORE, systematic coronary risk evaluation; VIPVIZA, Visualisation of asymptomatic atherosclerotic disease for optimum cardiovascular prevention.

#### Study outcome

Time to initiation of lipid-lowering agents and antihypertensives, defined as number of days, were identified within the time frame from inclusion in the VIPVIZA study until the study participants’ 3 year follow-up visit. This time frame was defined as the follow-up period. The initiation of either antihypertensives or lipid-lowering drug treatment was defined as a binary event (yes or no) and identified as the first drug dispensation of at least one of the specified ATC codes, respectively.

### Patient and public involvement

None.

### Ethics approval

The VIPVIZA study is registered with ClinicalTrials.gov, number NCT01849575. The Regional Ethical Review Board in Umeå has approved the study (2011-445-31M, amendments 2013-373-32M, 2016-245-32M, 2017-95-32M). Informed consent was obtained from all individual participants included in the study.

### Data sources

From the VIPVIZA study database, information on sex, age, other baseline characteristics, time for inclusion in the VIPVIZA study as well as BMQ were collected.^7^

The BMQ is divided into two blocks measuring the general beliefs about medicines with the dimensions overuse, harm and benefit, used in this study (presented in online supplemental appendix 3), and the specific beliefs about a participant’s own medicines with dimensions necessity and concern. BMQ is composed of statements with Likert scale measuring level of agreement from 1 to 5. Accordingly, the participants’ perception that drugs in general are overused, harmful and beneficial is scored 0–20 for each subscale. A higher score indicates a stronger perception as defined in the BMQ questionnaire.^16^

In the Swedish Prescribed Drug Register, all drug prescriptions dispensed at Swedish pharmacies are included. For this study, information on dispensed antihypertensive and lipid-lowering drugs within 6 months before inclusion in the VIPVIZA study as well as during the time period until the participants’ 3 year follow-up was extracted.

### Statistical analyses

Baseline characteristics and results are presented as frequencies (percentages), mean values with standard deviation (SD) and median with interquartile range (IQR) when suitable.

Cox-regression analyses, adjusted for sex, were conducted to investigate how the three general beliefs about medicines affect time to the initiation of antihypertensive or lipid-lowering drug treatment, from inclusion in the VIPVIZA study until the participants’ 3 year follow-up visit. The time to initiation was treated as a continuous variable, and the initiation of drug treatment was treated as a dichotomous variable. An assumption was made that beliefs about medicines are stable over time.^14^ Adjustment was made for sex as it has been found that adherence differs between men and women.^25^,^27^ No adjustments for CVD risk factors were made as no information about IMT or plaque were available for the control group at baseline and because CVD risk factors did not differ between the intervention and control group.^7^ BMQ was utilised as a continuous variable, that is, an assumption was made that the same effect was seen over every step on the BMQ scale. To check this assumption, the same Cox regression analyses were conducted using BMQ as a categorical variable, divided into four groups (data not shown). Moreover, the proportional hazards assumption of the Cox regression model was assessed using Schoenfeld residuals. Stratified analyses were performed for the VIPVIZA intervention and control group, separately, as the intervention has been found to affect drug initiation.^10^ All statistical calculations were conducted using IBM SPSS Statistics (Armonk, NY, USA) version 28 and R version 4.3.2.^28^

## Results

Of the 3532 participants included in the VIPVIZA, 89.7% (n=3167) participated in the 3 year follow-up and 73.1% (n=2314) received the BMQ of whom 97.6% (n=2258) had complete answers to at least one subscale in BMQ general. No significant difference was found between the control and intervention group regarding their beliefs about medicines being overused, harmful or beneficial (results shown in online supplemental appendix 4).

### General beliefs about medicines and the initiation of antihypertensive drugs

Around 15% of both control- and intervention-group participants dispensed antihypertensive medication. Participants included in the VIPVIZA control group, who reported stronger negative beliefs about medicines being overused, had a significantly lower rate of initiation of antihypertensive drug treatment (HR 0.91; 95% CI 0.84 to 0.996) compared with those with lower scores and consequently weaker negative perceptions. No statistically significant effect was found among the intervention-group participants (HR 1.05; 95% CI 0.95 to 1.16), shown in table 2.

**Table 2:** Effect on time to initiation of antihypertensive treatment in people with perception of overuse, harm and benefit according to the BMQ subscales and sex is presented as adjusted HR with 95% CI based on Cox regression model. Analyses are stratified for the control and intervention group, respectively

	Overuse				Harm				Benefit			
	Control (events=71)		Intervention (events=58)		Control (events=71)		Intervention (events=60)		Control (events=71)		Intervention (events=60)	
	n	HR (95% CI)	n	HR (95% CI)	n	HR (95% CI)	n	HR (95% CI)	n	HR (95% CI)	n	HR (95% CI)
BMQ*	449	0.91 (0.84–0.996)	429	1.05 (0.95–1.16)	444	0.97 (0.89–1.05)	433	1.04 (0.94–1.14)	450	1.01 (0.91–1.14)	433	0.98 (0.87–1.12)
Sex												
Men	173	Ref.	191	Ref.	170	Ref.	190	Ref.	172	Ref.	190	Ref.
Women	276	0.91 (0.57–1.47)	238	0.80 (0.48–1.34)	274	0.91 (0.57–1.46)	243	0.84 (0.50–1.39)	278	0.92 (0.57–1.47)	243	0.82 (0.49–1.36)

* Score range 4–20, 20=strongly believes, on all four questions within each subscale, that drugs are overused, harmful or beneficial. Events=Number of participants initiating antihypertensive treatment in each group.

BMQ, Beliefs about medicines questionnaire; CI, confidence interval; HR, hazard ratio.

Regardless of how strong the perception about medicines being harmful or beneficial was among the control- and intervention-group participants, respectively, there was no significant association among those with stronger beliefs about the harmfulness or the benefits and time to initiation of antihypertensive drugs (table 2). Moreover, no effect on time to initiation was found for sex.

### General beliefs about medicines and the initiation of lipid-lowering drugs

Around 10% in the control group and 23% in the intervention group dispensed lipid-lowering medication during the observation period. Regardless of participants’ perception about medicines being overused, harmful or beneficial, this was not associated with time to initiation of lipid-lowering drugs. Data are shown in table 3. Notably, a non-significant trend was observed, indicating longer time to initiation of lipid-lowering drugs among women when compared with men, in the control group but not in the intervention group.

**Table 3:** Effect on time to initiation of lipid-lowering treatment in people with perception of overuse, harm and benefit according to the BMQ subscales and sex is presented as adjusted HR with 95% CI based on Cox regression model. Analyses are stratified for the control and intervention group, respectively

	Overuse				Harm				Benefit			
	Control (events=48)		Intervention (events=132)		Control (events=48)		Intervention (events=131)		Control (events=48)		Intervention (events=131	
	n	HR (95% CI)	n	HR (95% CI)	n	HR (95% CI)	n	HR (95% CI)	n	HR (95% CI)	n	HR (95% CI)
BMQ*	604	0.94 (0.85–1.04)	564	0.97 (0.91–1.04)	600	0.96 (0.87–1.07)	568	1.01 (0.95–1.08)	606	0.96 (0.83–1.09)	570	1.00 (0.92–1.09)
Sex												
Men	247	Ref.	264	Ref.	244	Ref.	261	Ref.	248	Ref.	263	Ref.
Women	357	0.63 (0.36–1.10)	300	1.02 (0.72–1.43)	356	0.68 (0.39–1.19)	307	1.01 (0.71–1.42)	358	0.63 (0.36–1.11)	307	0.98 (0.69–1.38)

* Score range 4–20. 20=strongly believes, on all four questions within each subscale, that drugs are overused, harmful or beneficial. Events=Number of participants initiating lipid-lowering treatment in each group.

BMQ, Beliefs about medicines questionnaire; CI, confidence interval; HR, hazard ratio.

## Discussion

Cardiovascular preventive medications are effective and well proven but still underused.^3^,^6^ The VIPVIZA study showed that the intervention increased the initiation of lipid-lowering drugs markedly in the intervention group^10^ and decreased the cardiovascular risk in participants up to 3 years.^8^ The present study contributes to understanding of the connection between beliefs about medicines and the use of cardiovascular preventive medications within the VIPVIZA intervention, giving important information on how to further improve cardiovascular preventive strategies. Perceptions that medicines in general are being overused were found to significantly affect the time to initiation of antihypertensive drug treatment among participants included in the VIPVIZA control group; however, this effect was not found among participants in the intervention group. Since no significant difference was found between the control and intervention groups regarding their beliefs about medicines, this supports that the VIPVIZA intervention, with pictorial information, general information about atherosclerosis and follow-up phone call counselling, bridges negative perceptions about medicines being overused and, accordingly, affects the initiation of antihypertensive medications in a positive way.

Repeated information is beneficial to improve drug initiation,^10^ and multifactorial information is recommended to prevent cardiovascular disease.^25^ Even if general beliefs about medicines are reported to be stable over time,^14^ it has been found that interventions, for example, telephone counselling, moderately affected the perception of medicines.^15^ This further supports that the multi-factorial VIPVIZA intervention can affect beliefs about medicines and accordingly the willingness to initiate cardiovascular preventive medications.

It has been reported a negative perception among patients and among physicians regarding the use of lipid-lowering drugs as primary prevention among low-risk patients.^29^ However, the perception of medicines being overused, harmful or beneficial did not significantly affect time to initiation of lipid-lowering drugs in the control nor in the intervention group in the present study.

The intervention included information to the physicians that the presence of plaque indicates very high risk of CVD, which contributed to prescribing of lipid-lowering drugs among the doctors.^9^ Moreover, the intervention resulted in more knowledge about atherosclerosis and higher motivation to CVD prevention among the intervention-group participants and also enabled shared decision-making^30^ and improved risk perception.^23^ Additionally, guidelines state that lipid-lowering drugs should be prescribed when plaques are present,^2^ but there are no guidelines which recommend antihypertensive drugs in these situations, which explains the different proportions of lipid-lowering and antihypertensive drug treatment, respectively. Altogether, this indicates that the indication for lipid-lowering treatment, when plaques are present, has stronger influence on the initiation of lipid-lowering treatment than the participants’ general beliefs about medicines. Previous studies state that men have better adherence^26^ to lipid-lowering drug treatment compared with women.^25 27^ In the present study, we could see the same trend, but it was non-significant.

A limitation with the present study is that not all participants received the BMQ questionnaire and only at the 3 year follow-up. It is therefore not possible to compare the BMQ result with baseline and accordingly not possible to identify to what extent the VIPVIZA intervention affected the general beliefs about medicines in the intervention group, from baseline to the 3 year follow-up. Strengths are the high response rate and the validated BMQ questionnaire which was used in the population-based PROBE trial reflecting the clinical reality in a population at low/intermediate risk of CVD.

## Conclusions

The perception of drugs being overused significantly affects the willingness to initiate antihypertensive drug treatment in a negative manner. The result in the present study suggests that the VIPVIZA intervention overbridges this negative perception about medicines and positively affects the initiation of antihypertensive medications.

## Supplementary material

10.1136/bmjopen-2025-100924 online supplemental file 1

10.1136/bmjopen-2025-100924 online supplemental file 2

10.1136/bmjopen-2025-100924 online supplemental file 3

10.1136/bmjopen-2025-100924 online supplemental file 4

## Data Availability

Data are available upon reasonable request.
