# Supplementary material for: Beliefs about medicines in relation to the initiation of cardiovascular preventive medications during a 3 year follow-up period after inclusion in the VIPVIZA trial: a cohort study
Source: BMJ Open. 2025 Dec 23;15(12):e100924. doi: 10.1136/bmjopen-2025-100924 (PMC12730765; doi:10.1136/bmjopen-2025-100924)
Supplement: online supplemental file 2 [file bmjopen-15-12-s002.pdf]

Your carotid wall thickness, IMT, is illustrated as vascular age

Your value is shown in comparison to people of the same sex and age.
The pointer goes from smaller to larger wall thickness.
Green sector corresponds to the wall thickness of people who are at least 10 years younger than you, red sector who are at least 10 years older.

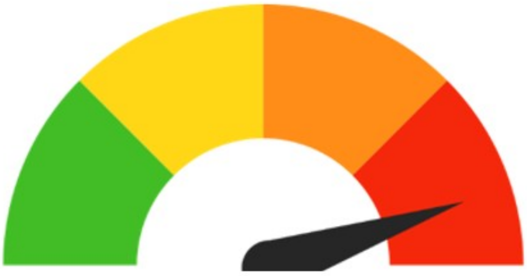

Right side	Left side
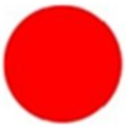	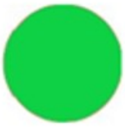
PLAQUE DETECTED	PLAQUE NOT DETECTED
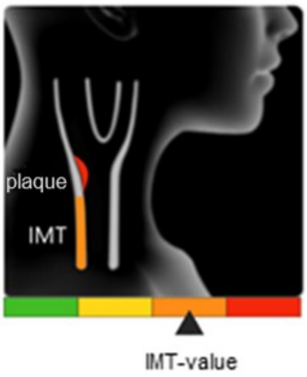	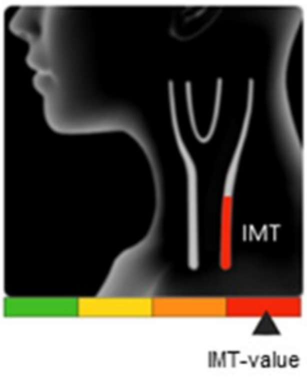
