# Supplementary material for: Beliefs about medicines in relation to the initiation of cardiovascular preventive medications during a 3 year follow-up period after inclusion in the VIPVIZA trial: a cohort study
Source: BMJ Open. 2025 Dec 23;15(12):e100924. doi: 10.1136/bmjopen-2025-100924 (PMC12730765; doi:10.1136/bmjopen-2025-100924)
Supplement: online supplemental file 3 [file bmjopen-15-12-s003.pdf]

Supplementary material

Beliefs about medicines in relation to initiation of cardiovascular preventive medications during a three-year follow up period after inclusion in the VIPVIZA trial: a cohort study

BMJ Open

Eva Sönnerstam [a], Henrik Holmberg [b], Bo Carlberg [c], Margareta Norberg [c], Anders Själander [c], Eva-Lotta Glader [c]

[a] Department of Medical and Translational Biology, Umeå University, 901 87 Umeå, Sweden

[b] Department of Epidemiology and Global Health, Umeå University, 901 87 Umeå, Sweden

[c] Department of Public Health and Clinical Medicine, Umeå University, 901 87 Umeå, Sweden

E-mail:

eva.sonnerstam@umu.se

henrik.holmberg@umu.se

bo.carlberg@umu.se

margareta.norberg@umu.se

anders.sjalander@umu.se

eva-lotta.glader@umu.se

Corresponding Author:

Eva Sönnerstam, eva.sonnerstam@umu.se

The beliefs about medicines questionnaire (BMQ) general

Subscale – Overuse

- Doctors prescribe too many medications.
- Natural remedies are safer than traditional drug treatment.
- Doctors trust medications too much
- If doctors had more time with their patients, they would prescribe fewer drugs.

Subscale – Harm

- People who use medications should paus their treatment now and then.
- Most medications are additive.
- All medications are poisons.
- Medications do more harm than good.

Subscale – Benefit

- Medications help a many people to live better lives.
- The benefit with medications outweighs the risks in most of the cases.
- Without medications, doctors would have fewer possibility to cure people.
- Medications help many people to live longer.
