# Supplementary material for: Beliefs about medicines in relation to the initiation of cardiovascular preventive medications during a 3 year follow-up period after inclusion in the VIPVIZA trial: a cohort study
Source: BMJ Open. 2025 Dec 23;15(12):e100924. doi: 10.1136/bmjopen-2025-100924 (PMC12730765; doi:10.1136/bmjopen-2025-100924)
Supplement: online supplemental file 4 [file bmjopen-15-12-s004.pdf]

Supplementary material

Beliefs about medicines in relation to initiation of cardiovascular preventive medications during a three-year follow up period after inclusion in the VIPVIZA trial: a cohort study

BMJ Open

Eva Sönnerstam [a], Henrik Holmberg [b], Bo Carlberg [c], Margareta Norberg [c], Anders Själander [c], Eva-Lotta Glader [c]

[a] Department of Medical and Translational Biology, Umeå University, 901 87 Umeå, Sweden

[b] Department of Epidemiology and Global Health, Umeå University, 901 87 Umeå, Sweden

[c] Department of Public Health and Clinical Medicine, Umeå University, 901 87 Umeå, Sweden

E-mail:

eva.sonnerstam@umu.se

henrik.holmberg@umu.se

bo.carlberg@umu.se

margareta.norberg@umu.se

anders.sjalander@umu.se

eva-lotta.glader@umu.se

Corresponding Author:

Eva Sönnerstam, eva.sonnerstam@umu.se

Table S1. Comparison between the control and intervention group regarding their beliefs about medicines, using independent samples t-test.

**BMQ subscale**[a]	**Control** (mean (SD))	**Intervention (**mean (SD))	**p-value**
Overuse[b]	11.8 (2.6)	11.7 (2.6)	0.53
Harm[c]	9.3 (2.7)	9.2 (2.7)	0.43
Benefit[d]	16.9 (2.0)	16.7 (2.1)	0.08

[a] Score range 4-20
[b] Control (n=1131), Intervention (n=1103)
[c] Control (n=1125), Intervention (n=1104)
[d] Control (n=1137), Intervention (n=1108)
