# Supplementary material for: Beliefs about medicines in relation to the initiation of cardiovascular preventive medications during a 3 year follow-up period after inclusion in the VIPVIZA trial: a cohort study
Source: BMJ Open. 2025 Dec 23;15(12):e100924. doi: 10.1136/bmjopen-2025-100924 (PMC12730765; doi:10.1136/bmjopen-2025-100924)
Supplement: Supplementary file 5 [file bmjopen-2025-100924.reviewer_comments.pdf]

PEER REVIEW HISTORY

BMJ Open publishes all reviews undertaken for accepted manuscripts. Reviewers are asked to complete a checklist review form and are provided with free text boxes to elaborate on their assessment. These free text comments are reproduced below.

ARTICLE DETAILS

Title (Provisional)

Beliefs about medicines in relation to initiation of cardiovascular preventive medications during a three-year follow-up period after inclusion in the VIPVIZA trial: a cohort study

Authors

Sönnerstam, Eva; Holmberg, Henrik; Carlberg, Bo; Norberg, Margareta; Själander, Anders; Glader, E-L

VERSION 1 - REVIEW

**Reviewer**	**1**
**Name**	**Dewi, Ayu Putu Madri**
**Affiliation**	**Amsterdam UMC Location AMC, Epidemiology and Data Science**
**Date**	**05-Mar-2025**
**COI**	**None**

I am an Open Science researcher, who has checked your manuscript for its adherence to a number of Open Science practices.

Below you find the checklist that I used to assess your manuscript for adherence to a number of these practices.

Based on this assessment, I would suggest the following refinements/additions:

1. *Availability of a protocol: OK*
2. *Pre-registration: OK*
3. *Open Materials: OK*
4. *Open Data: Given the confidentiality of the data, please specify whether de-identified, metadata, or a subset of the data can still be made publicly available.*
5. *Open Code: Please provide (a link to) the code used for the analyses in this study.*
6. *Citations: Please provide citations for your materials/data/codes.*
7. *Software and tools used are described: OK*
8. *Open access publication: OK*
9. *Reporting checklist provided: OK*
10. *Preprint: Please state explicitly whether and where the preprint of this study can be found.*

**Reviewer**	**2**
**Name**	**Ho, Chau**
**Affiliation**	**Curtin University Bentley Campus**
**Date**	**05-May-2025**
**COI**	**None**

The manuscript was interesting to read. I have some comments as below:

1. Introduction: what is the rational of examining the association with CVD drug initiation? particularly for those with low/ moderate risk? what are the indications or guideline recommendation for CVD drug initiation in those with low/ moderate CVD risk?

2. Methods

- what is the study design?

-The authors reported the inclusion crieteria in the VIPVIZA study. However it is unclear what were the inclusion and exclusion criteria of the current study? are they the same with the VIPVIZA study?

- how was BMQ scale defined for perception of oversuse, harm and benefit drug use?

- the BMQ was measured at the 3-year followup visit, outcomes were measured during the 3-year followup. if BMQ scale is the main exposure, the main exposure seemed to be measured after the occurence of the outcome -> how was cox-regression model set up?

- time to initiation of antihypertensive was continuous (number of days) ? how was the follow-up period defined? (e.g. endpoint - initiation of CVD drugs, death, censoring....)

- Was there any adjustment for covariates/confounders ??

- Was there any adjustment for p value when there is multiple comparison? p for interaction for subgroup analysis?

3. Results:

- it will be informative to have a table of baseline characteristics between groups of different perceptions on drug use.

- the presentation of the results in table 2 and 3 is a bit confusing.

**Reviewer**	**3**
**Name**	**Dahl, Marie**
**Affiliation**	**Regionshospitalet Viborg, Cardiovascular Research Unit**
**Date**	**19-Aug-2025**
**COI**	**None**

This study addresses an important topic, as lack of adherence to preventive treatment is a well-known challenge, particularly among patients who are asymptomatic. By incorporating the Beliefs about Medicines Questionnaire (BMQ), the study provides valuable insights into the perspectives of screening participants and explores whether the BMQ can serve as a useful tool for identifying factors associated with prescription uptake. As adherence to preventive recommendations is essential for the effectiveness of any preventive intervention, this study provides an important contribution to the literature on cardiovascular prevention.

I appreciate having had the opportunity of reviewing this article. I hope you will find my comments both constructive and helpful. That was my goal.

Abstract

Overall, the abstract aligns with the structure given by BMJOpen. However, the text would benefit from a revision to optimise sentence structure and avoid repetitions. In addition, abbreviations should be written out in full at first mention e.g. BMQ and VIPVIZA.

Setting:

Please add country.

Participants:

An example of An example of where the structure could be optimised is the following sentence: “People participating in the VIPVIZA three-year follow-up (n=3,167 (89.7%)), receiving the BMQ at the three-year follow-up (n=2,314 (73.1%))”. The phrase “three-year follow-up” appears twice, which reduces readability.

Measures:

The authors are recommended to add the used statistical approach used for the primary outcome e.g. Cox proportional hazards models, presented as unadjuseted or adjusted, and if adjusted, which covariates were included in the models.

Conclusion:

The sentence "significantly affect the willingness" may be rephrased for clarity and to align with the objectives. Perhaps: “The perception that medicines are overused was associated with delayed initiation of antihypertensive treatment”

Strengths and limitations of the study

Appropriate – no comments.

Introduction:

The authors need to add references on their statement: “Cardiovascular preventive medications are effectives and proven but still underused” (page 5)

Methods

In the study population section, the inclusion criteria for the current study needs to be stated (page 6).

The authors need to add references on their statement: “Written information was also given about atherosclerosis being a dynamic process that potentially can be reduced, or even reversed, through healthier lifestyle habits and, if indicated, preventive pharmacological treatment” (page 6 lines 56-59).

The authors need to add reference for the guideline stating that carotid plaque indicate a very high risk of CVD.

For clarity, it would be helpful if the authors specify how carotid plaque was defined in your study, as definitions of plaque vary considerably in the carotid plaque literature.

The ATC abbreviation is need to be written in full the first time.

Statistical analysis

The authors need to add the test(s) used for presenting baseline characteristics.

Did the authors assess the proportional hazards assumption of the Cox regression model, for example by using log–log plots and Schoenfeld residuals?

In the Cox regression analyses, adjustment was made for sex. Please provide the rationale for this choice.

Results

The statement “data not shown” regarding the balance between control and intervention groups makes it difficult for readers to assess comparability. The authors are recommend to provide these data in the supplementary material instead.

Since initiation of cardiovascular preventive medication is likely to be related to the presence or absence of carotid plaque, this could represent an interesting sub-analysis, similar to the sex-stratified analysis in Table 2 and 3. Have the authors considered conducting such sub-analyses?

Discussion

The authors need to add references on their statement: “Cardiovascular preventive medications are effectives and proven but still underused” (page 12)

The structure of paragraph 2 is not fully consistent with academic argumentation. I suggest revising it using a clearer argumentative framework, for example by applying Toulmin’s model of argumentation (page 12, lines 30-36).

The authors states that guidelines recommend initiation of lipid-lowering therapy when carotid plaque is present. A reference is required to support this claim.

Tables

Fine. Perhaps add whether the HR is unadjusted or adjusted.

VERSION 1 - AUTHOR RESPONSE

Reviewer: 1
Ms. Ayu Putu Madri Dewi, Amsterdam UMC Location AMC Comments to the Author:
I have some feedback related to open science practices. Please find attached the feedback details.

I am an Open Science researcher, who has checked your manuscript for its adherence to a number of Open Science practices.

Below you find the checklist that I used to assess your manuscript for adherence to a number of these practices.

Based on this assessment, I would suggest the following refinements/additions:

1. **Availability of a protocol: OK**

2. **Pre-registration: OK**

3. **Open Materials: OK**

4. **Open Data: Given the confidentility of the data, please specify whether de-identified, metadata, or a subset of the data can still be made puclicly available.**

   ⇨ Thank you for this relevant comment. As the deidentified data underlying the research article, were provided to the research group by permission from VIPVIZA according to Swedish regulations and the Swedish Ethical Review Authority it is not possible to make deidentified, metadata or a subset of the data publicly available. The following sentence is added in the "Data availability statement" on page 16 to inform the reader about this:

Accordingly, neither deidentified data, metadata nor a subset of the data can be publicly available.

   ⇨ Also, to inform the reader who to contact about data sharing, the following sentence in the "Data availability statement" on page 16-17 is revised from:

Data will be shared on reasonable request to VIPVIZA and can only be used according to the purposes given in the ethical approval given by the Swedish Ethical Review Authority.

To:

Data will be shared on reasonable request to PI Patrik Wennberg (Email: patrik.wennberg@umu.se) and can only be used according to the purposes given in the ethical approval given by the Swedish Ethical Review Authority.

5. **Open Code: Please provide (a link to) the code used for the analyses in this study.**

   a. Thank you for bringing this to our attention. No syntax was used when the statistical analyses were conducted in SPSS. Accordingly, no open code is available. The following information is added as the last sentence in the "Data availability statement" on page 17:

Open code is unavailable as syntax was not utilized in the statistical analyses.

6. **Citations: Please provide citations for your materials/data/codes.**

   a. Thank you for noticing this. Citations are now added in the "Data collection" paragraph, and the following citation is added after the first sentence on page 7:

For both groups information on risk factors were collected from the VIP examination at baseline (7).

   b. Näslund U, Ng N, Lundgren A, Fhärm E, Grönlund C, Johansson H, et al. Visualization of asymptomatic atherosclerotic disease for optimum cardiovascular prevention (VIPVIZA): a pragmatic, open-label, randomised controlled trial. Lancet. 2019;393(10167):133-4

The following citation is added after the second sentence on page 7:

The 3-year follow-up examinations were performed between September 2016 and May 2019 (8).

Bengtsson A, Norberg M, Ng N, Carlberg B, Grönlund C, Hultdin J, et al. The beneficial effect over 3 years by pictorial information to patients and their physician about subclinical atherosclerosis and cardiovascular risk: Results from the VIPVIZA randomized clinical trial. Am J Prev Cardiol. 2021;7:100199.

The following citation is added after the fourth sentence on page 8:

In March 2017, the BMQ was added to the VIPVIZA questionnaire (16).

16. Horne R, Weinman J, Hankins M. The beliefs about medicines questionnaire: The development and evaluation of a new method for assessing the cognitive representation of medication. Psychology & Health. 1999;14(1):1-24.

Also, on page 11, in the "Statistical analyses" section, second paragraph, last sentence, SPSS and R are cited as follows:

All statistical calculations were conducted using SPSS version 28 and R version 4.3.2 (28).

To further clarify for the reader. This sentence is revised to:

All statistical calculations were conducted using IBM SPSS Statistics (Armonk, NY, USA) version 28 and R version 4.3.2 (28).

7. **Software and tools used are described: OK**

8. **Open access publication: OK**

9. **Reporting checklist provided: OK**

10. **Preprint: Please state explicitly whether and where the preprint of this study can be found.**

⇨ Thank you for noticing this. I order to clarify, the following title and sentence are added on page 17, after the "Data availability statement":

Preprint
This study has not been published as a preprint.

Reviewer: 2
Dr. Chau Ho, Curtin University Bentley Campus
Comments to the Author:
The manuscript was interesting to read. I have some comments as below:
1. **Introduction**

What is the rational of examining the association with CVD drug initiation?

⇨ Thank you for highlighting this. We agree that the rational for the study can be further clarified and the following sentence, in the last paragraph in the "Introduction" on page 6, is revised from:

The association between the patients' beliefs about medicines and use of preventive cardiovascular drugs has been more extensively studied in relation to drug adherence, but the association with drug initiation is less explored (12).

To:

The association between the patients' beliefs about medicines and use of preventive cardiovascular drugs has been more extensively studied in relation to drug adherence in terms of implementation, but the association with drug initiation, i.e. the first step in the adherence process, is less explored (12).

Additionally, the following sentences and a supporting reference are added before the study aim, in the last paragraph, on page 6:

Moreover, if patients' beliefs about medicines affects how fast they initiate recommended CVD- preventive medications is unknown. Since the effect of drug treatment is dependent on adherence (17) it is important to gain further knowledge about this process to further improve cardiovascular preventive strategies.

Particularly for those with low/ moderate risk?

⇨ The VIPVIZA-study mainly included those with low/moderate risk of CVD as this risk group has been sparingly investigated in previously published RCT's even if this group of people represents 60-70% of all cardiovascular disease events. It is therefore crucial to improve primary

CVD prevention to further lower the prevalence CVD morbidity and mortality among this risk group. To further highlight this, the following information together with a reference is added as the second sentence in the first paragraph on page 5 in the “Introduction”:

Among all cardiovascular disease events, 60-70% are presented among people considered being at low or moderate risk for CVD, categorized according to the risk assessment model Systemic Coronary Risk Estimation (SCORE) (2) and updated SCORE 2 (3).

- ⇨ Moreover, those with significant stenosis, defined as more than 50% luminal narrowing based on the vascular ultrasound, were excluded from the VIPVIZA-study and directly referred to specialised care as stated in the following sentence in the first paragraph in the “Methods” section on page 7:

People with severe carotid stenosis according to the vascular ultrasound at baseline were excluded from the study and referred to specialized care directly (n=22 out of 4,177 screened).

What are the indications or guideline recommendation for CVD drug initiation in those with low/ moderate CVD risk?

- ⇨ This is a relevant question. Participants categorized as having low/moderate CVD risk were categorized as such according to SCORE, i.e. the 10-year risk of fatal CVD based on age, sex, smoking, systolic blood pressure and total cholesterol. However, other risk factors may increase their risk of CVD, as included in the inclusion criteria in the VIPVIZA study, and as stated in the European guidelines, i.e. the participants total risk may be higher than the SCORE classification if also being e.g. sedentary or being obese, having diabetes, familial hypercholesterolemia and/or increased IMT and/or plaque detected by carotid ultrasonography. Accordingly, individuals with low/moderate CVD risk may be subject for antihypertensive and lipid-lowering drug initiation based on the total cardiovascular risk and their blood-pressure and cholesterol levels. To inform the reader about this the following sentence is added in the first paragraph in the “Introduction” on page 5:

The treatment decision should be based on SCORE/SCORE 2 as well as the total cardiovascular risk (2, 3).

Methods

- What is the study design?

- ⇨ Thank you for this comment. The study design is now added on page 6, “Study population and definitions”. The first sentence in this is paragraph is changed from:

The participants in the present study, described according to Strengthening the Reporting of Observational Studies in Epidemiology (STROBE) cohort reporting guidelines (18), participated in the Pragmatic Randomized Open Blinded End-point (PROBE) VIPVIZA trial, which included mainly people at low/medium risk of CVD (7).

To:

The participants in the present questionnaire and register-based cohort study, described according to Strengthening the Reporting of Observational Studies in Epidemiology (STROBE) cohort reporting guidelines (18), participated in the Pragmatic Randomized Open Blinded End-point (PROBE) VIPVIZA trial, which included mainly people at low/medium risk of CVD (7).

-The authors reported the inclusion criteria in the VIPVIZA study. However it is unclear what were the inclusion and exclusion criteria of the current study? are they the same with the VIPVIZA study?

- ⇨ Thank you for these questions. The present study includes the VIPVIZA participants, however, further inclusion criteria was added in the present study. We agree that the inclusion criteria can be further clarified as they are now included in the ”Data collection” paragraph. Accordingly, we have made the following revisions in the manuscript:

The title ”*Inclusion criteria”* is added on page 8 after the following sentence:

Accordingly, there was no selection of participants receiving the BMQ questionnaire and baseline characteristics did not differ significantly, neither between people who received the questionnaire and those who did not, nor between those with full response and those with at least one missing on the BMQ (data not shown).

Moreover, the first sentence following the “Inclusion criteria” title on page 8 is revised from:

To avoid confounding due to different inclusion criteria by age, only participants who were 60-years old were included in the current study.

To:

To avoid confounding due to different inclusion criteria by age, only participants from the control and intervention groups who were 60-years old were included in the current study.

Also, to further clarify the inclusion criteria, the penultimate sentence in the "Inclusion criteria" paragraph, on page 8, is rewritten. From:

A flow chart over the study population is shown in Figure 1.

To:

A flow chart over the study population in the present study is shown in Figure 1.

- how was BMQ scale defined for perception of overuse, harm and benefit drug use?

- ⇨ Thank you for asking this important question. This information should of course be included in the manuscript, and the following sentence is therefore added last in the second paragraph in the "Data sources" section on page 10:

Accordingly, the participants perception that drugs in general are overused, harmful and beneficial are scored 0 – 20 for each subscale. A higher score indicates a stronger perception as defined in the BMQ questionnaire (16).

- the BMQ was measured at the 3-year follow-up visit, outcomes were measured during the 3-year follow-up. if BMQ scale is the main exposure, the main exposure seemed to be measured after the occurrence of the outcome -> how was cox-regression model set up?

- ⇨ This is a relevant question, and it is correct that the main exposure, i.e. the BMQ scale, is measured after the occurrence of the outcome. Consequently, we assume that BMQ, and accordingly the exposure, is stable and do not change between baseline, i.e. the start of the 3-year follow up, and the 3-year follow up when beliefs about medicines was measured. This assumption is based on the following statement in the "Introduction" on page 5, paragraph four:

Irrespective of changes in health status, general beliefs about medicines have been shown stable over time (14).

The set up of the Cox-regression models are based on this assumption and the following sentence is now added into the second paragraph under the title "Statistical analyses" on page 11 to make the reader aware of this:

An assumption was made that beliefs about medicines are stable over time (14).

- time to initiation of antihypertensive was continuous (number of days) ?

- ⇨ It is correct that time to initiation of antihypertensive drugs, and also lipid lowering drugs, was a continuous variable, i.e. number of days as written in the first sentence under the title "Study outcome" on page 9:

Time to initiation of lipid-lowering agents and antihypertensives, defined as number of days, were identified within the time frame from inclusion in the VIPVIZA study until the study participants' three- year follow-up visit.

Moreover, to further clarify for the reader, the following sentence in the paragraph "Statistical analyses" on page 11 is changed from:

Cox-regression analyses, adjusted for sex, were conducted to investigate how the three general beliefs about medicines affect time to initiation of antihypertensive or lipid-lowering drug treatment, from inclusion in the VIPVIZA-study until the participants' three-year follow-up visit.

To:

Cox-regression analyses, adjusted for sex, were conducted to investigate how the three general beliefs about medicines affect time to initiation of antihypertensive or lipid-lowering drug treatment (treated as a continuous variable), from inclusion in the VIPVIZA-study until the participants' three-year follow-up visit.

how was the follow-up period defined? (e.g. endpoint - initiation of CVD drugs, death, censoring.......)

- ⇨ This is a relevant question. The follow up period was defined as the time frame between the individual participant's inclusion in the VIPVIZA study until the participant's 3-year follow up. During this timeframe the mortality rate are low. Only 13 people from the intervention group and 7 from the control group corresponding to 0.6% mortality in the total study population (n=3,532). Accordingly, the risk for death in the population is low. To clarify this information for the reader the following sentence is added in the "Study outcome" paragraph on page 9:

This time frame was defined as the follow-up period.

Moreover, the following sentence is added as the third sentence in the "Data collection" paragraph on page 7-8:

The mortality rate was only 13 participants in the intervention group and 7 participants in the control group corresponding to 0.6% of the total study population (8).

- Was there any adjustment for covariates/confounders ??

- ⇨ Yes, the statistical analyses were adjusted for sex as stated in the following sentence in the second paragraph on page 11:

Cox-regression analyses, adjusted for sex, were conducted to investigate how the three general beliefs about medicines affect time to initiation of antihypertensive or lipid-lowering drug treatment (treated as a continuous variable), from inclusion in the VIPVIZA-study until the participants' three-year follow-up visit.

- Was there any adjustment for p value when there is multiple comparison?
p for interaction for subgroup analysis?

- ⇨ This is relevant questions. As only one comparison was made when investigating the main outcome, we do not think that any adjustment for p-value should be made. Each analyse include one of the three subscales included in the BMQ general, i.e. one main scale. Therefore, we do not think that any interaction for subgroup analysis are needed either.

Results:
- it will be informative to have a table of baseline characteristics between groups of different perceptions on drug use.

- ⇨ Thank you for this interesting suggestion. However, as most of the participants answered all three subscales concerning general beliefs about medicines, i.e. overuse, harm and benefit, the baseline characteristics would be approximately the same for all three subgroups if there is a baseline table based on perceptions of drug use. We therefore think that presentation of baseline characteristics based on drug treatment is the most appropriate and transparent way to present the data.

- the presentation of the results in table 2 and 3 is a bit confusing.

- ⇨ Thank you for letting us know. As Reviewer 3 think that the presentation of results in table 2 and 3 are fine we decide to not revise either table 2 or 3. However, according to the comment from Reviewer 3 the table caption could be clarified. The first sentence in the table 2 caption, is therefore revised from:

Effect on time to initiation of antihypertensive treatment in people with perception of overuse, harm and benefit according to the BMQ subscales and sex are presented as HR with 95% CI based on Cox regression model.

To:

Effect on time to initiation of antihypertensive treatment in people with perception of overuse, harm and benefit according to the BMQ subscales and sex are presented as adjusted HR with 95% CI based on Cox regression model.

And in table 3, the first sentence in the caption is rewritten from:

Effect on time to initiation of lipid-lowering treatment in people with perception of overuse, harm and benefit according to the BMQ subscales and sex are presented as HR with 95% CI based on Cox regression model.

To:

Effect on time to initiation of lipid-lowering treatment in people with perception of overuse, harm and benefit according to the BMQ subscales and sex are presented as adjusted HR with 95% CI based on Cox regression model.

We hope that Reviewer agree with this suggestion and that these revisions clarify the result presentation. However, if Reviewer want us further to clarify, please let us know how this can be done.

Reviewer: 3
Mrs. Marie Dahl, Regionshospitalet Viborg
Comments to the Author:

This study addresses an important topic, as lack of adherence to preventive treatment is a well-known challenge, particularly among patients who are asymptomatic. By incorporating the Beliefs about Medicines Questionnaire (BMQ), the study provides valuable insights into the perspectives of screening participants and explores whether the BMQ can serve as a useful tool for identifying factors associated with prescription uptake. As adherence to preventive recommendations is essential for the effectiveness of any preventive intervention, this study provides an important contribution to the literature on cardiovascular prevention.

I appreciate having had the opportunity of reviewing this article. I hope you will find my comments both constructive and helpful. That was my goal.

Abstract
Overall, the abstract aligns with the structure given by BMJOpen.
However, the text would benefit from a revision to optimise sentence structure and avoid repetitions.

- ⇨ Thank you for your advice. We have revised the text in the Abstract according to your suggestion and think that the sentence structure has improved and repetitions has been removed. Accordingly, the following sentence in “Objectives” on page 2 has been changed from:

To investigate if beliefs about medicines affect the time to initiation of cardiovascular preventive medications during a three-year follow-up.

To:

To investigate if beliefs about medicines affect the time to initiation of cardiovascular preventive medications during a three-year follow-up period.

The following sentence after “Design” on page 2 has been changed from:

Questionnaire and register-based cohort study.

To:

A questionnaire and register-based cohort study.

The sentence after ”Setting” on page 2 has been rewritten from:

People, 40, 50 and 60 years old who underwent risk factor screening and individual health promotion within the Västerbotten intervention program (VIP) in primary care.

To:

Primary care in Sweden, in which people 40, 50 and 60 years old underwent risk factor screening and individual health promotion within the Västerbotten intervention program (VIP).

Finally, the full name of the BMQ is removed from the sentence within "Measures" section on page 2 to avoid repetition of already stated information. Accordingly, the following sentence is revised from:

General beliefs about medicines were assessed according to The Beliefs about Medicines Questionnaire (BMQ).

To:

General beliefs about medicines were assessed according to the BMQ.

In addition, abbreviations should be written out in full at first mention e.g. BMQ and VIPVIZA.

- ⇨ Thank you for noticing this. We agree and have written out in full at first mention of CVD, VIPVIZA and BMQ. Accordingly, the following sentences on page 2 in the "Abstract" are changed from:

People at low/medium risk of CVD according to the risk factor screening were included in the population-based Pragmatic Randomized Open Blinded End-point (PROBE) VIPVIZA trial, aiming at improved primary prevention of CVD. People participating in the VIPVIZA three-year follow-up (n=3,167 (89.7%)), receiving the BMQ at the three-year follow-up (n= 2,314 (73.1%)) and with complete answers to at least one subscale in the BMQ general (n = 2,258 (97.6%)) were included.

To:

People at low/medium risk of cardiovascular disease (CVD) according to the risk factor screening were included in the ***VI****sual****i****Z****ation of asymptomatic* ***A****therosclerotic disease for optimum cardiovascular prevention – a population-based Pragmatic Randomized Open Blinded End-point trial (PROBE) nested in the* ***V****ästerbotten* ***I****ntervention* ***P****rogramme (VIPVIZA), aiming at improved primary prevention of CVD. People participating in the VIPVIZA three-year follow-up (n=3,167 (89.7%)), receiving the Beliefs about medicines questionnaire (BMQ) (n= 2,314 (73.1%)) and with complete answers to at least one subscale in the BMQ general (n = 2,258 (97.6%)) were included.*

Setting:
Please add country.

- ⇨ Thank you for this suggestion. The following sentence on page 2 in the "Abstract" is now changed from:

Setting: *People, 40, 50 and 60 years old who underwent risk factor screening and individual health promotion within the Västerbotten intervention program (VIP) in primary care.*

To:

Setting: *Primary care in Sweden, in which people 40, 50 and 60 years old underwent risk factor screening and individual health promotion within the Västerbotten intervention program (VIP).*

Participants:
An example of where the structure could be optimised is the following sentence: "People participating in the VIPVIZA three-year follow-up (n=3,167 (89.7%)), receiving the BMQ at the three-year follow-up (n=2,314 (73.1%))". The phrase "three-year follow-up" appears twice, which reduces readability.

- ⇨ We agree that this sentence can be optimised according to your suggestion. The following sentence on page 2 is revised from:

People participating in the VIPVIZA three-year follow-up (n=3,167 (89.7%)), receiving the Beliefs about medicines questionnaire (BMQ) at the three-year follow-up (n= 2,314 (73.1%)) and with complete answers to at least one subscale in the BMQ general (n = 2,258 (97.6%)) were included.

To:

People participating in the VIPVIZA three-year follow-up (n=3,167 (89.7%)), receiving the Beliefs about medicines questionnaire (BMQ) (n= 2,314 (73.1%)) and with complete answers to at least one subscale in the BMQ general (n = 2,258 (97.6%)) were included.

Measures:
The authors are recommended to add the used statistical approach used for the primary outcome e.g. Cox proportional hazards models, presented as unadjusted or adjusted, and if adjusted, which covariates were included in the models.

⇨ Thank you for noticing this. The following sentence is now added on the fourth line in the "Measures" section on page 2:

Cox proportional hazards models, adjusted for sex, were conducted to investigate primary outcome.

Conclusion:
The sentence "significantly affect the willingness" may be rephrased for clarity and to align with the objectives. Perhaps: "The perception that medicines are overused was associated with delayed initiation of antihypertensive treatment"

⇨ We agree that your suggestion aligns better with the objectives and hope that you agree with the revision we have made. The following sentence on page 3 is changed from:

A more negative perception of drugs being overused significantly affect the willingness to initiate antihypertensive drug treatment.

To:

A more negative perception of drugs being overused was significantly associated with delayed initiation of antihypertensive drug treatment.

Strengths and limitations of the study Appropriate – no comments.

Introduction:
The authors need to add references on their statement: "Cardiovascular preventive medications are effectives and proven but still underused" (page 5)

⇨ Thank you for recognising this. The following four references are added to support the statement in the first paragraph on page 5:

3. Visseren FLJ, Mach F, Smulders YM, Carballo D, Koskinas KC, Bäck M, et al. 2021 ESC Guidelines on cardiovascular disease prevention in clinical practice. Eur J Prev Cardiol. 2022;29(1):5-115.

4. Kotseva K, De Bacquer D, De Backer G, Rydén L, Jennings C, Gyberg V, et al. Lifestyle and risk factor management in people at high risk of cardiovascular disease. A report from the European Society of Cardiology European Action on Secondary and Primary Prevention by Intervention to Reduce Events (EUROASPIRE) IV cross-sectional survey in 14 European regions. Eur J Prev Cardiol. 2016;23(18):2007-18.

5. Bradley CK, Wang TY, Li S, Robinson JG, Roger VL, Goldberg AC, et al. Patient-Reported Reasons for Declining or Discontinuing Statin Therapy: Insights From the PALM Registry. J Am Heart Assoc. 2019;8(7): e011765.

6. März W, Dippel FW, Theobald K, Gorcyca K, Iorga S, Ansell D. Utilization of lipid-modifying therapy and low-density lipoprotein cholesterol goal attainment in patients at high and very-high cardiovascular risk: Real-world evidence from Germany. Atherosclerosis. 2018;268:99-107.

Accordingly, the reference list is also renumbered.

Methods
In the study population section, the inclusion criteria for the current study needs to be stated (page 6).

⇨ We agree that this can be clarified as this information is included in the "Data collection" paragraph. Accordingly, the title "*Inclusion criteria"* is added on page 8 after the following sentence:

Accordingly, there was no selection of participants receiving the BMQ questionnaire and baseline characteristics did not differ significantly, neither between people who received the questionnaire and those who did not, nor between those with full response and those with at least one missing on the BMQ (data not shown).

Moreover, the first sentence following the “Inclusion criteria” title on page 8 is revised from:

To avoid confounding due to different inclusion criteria by age, only participants who were 60-years old were included in the current study.

To:

To avoid confounding due to different inclusion criteria by age, only participants from the control and intervention groups who were 60-years old were included in the current study.

Also, to further clarify the inclusion criteria, the last sentence in the “Inclusion criteria” paragraph, on page 8, is rewritten. From:

A flow chart over the study population is shown in Figure 1.

To:

A flow chart over the study population in the present study is shown in Figure 1.

The authors need to add references on their statement: “Written information was also given about atherosclerosis being a dynamic process that potentially can be reduced, or even reversed, through healthier lifestyle habits and, if indicated, preventive pharmacological treatment” (page 6 lines 56-59).

- ⇨ Thank you for this relevant comment. We have now added the following references on page 7 after this sentence in order to support this statement:

  2. Perk J, De Backer G, Gohlke H, et al. European Guidelines on cardiovascular disease prevention in clinical practice (version 2012). The Fifth Joint Task Force of the European Society of Cardiology and Other Societies on Cardiovascular Disease Prevention in clinical Practice (constituted by representatives of nine societies and by invited experts). Developed with the special contribution of the European Association for Cardiovascular Prevention & Rehabilitation (EACPR). Eur Heart J 2012; 33: 1635-701.

22. Athyros VG, Katsiki N, Karagiannis A, Mikhailidis DP. High-intensity statin therapy and regression of coronary atherosclerosis in patients with diabetes mellitus. J Diabetes Complications. 2015;29(1):142-5.

The authors need to add reference for the guideline stating that carotid plaque indicate a very high risk of CVD.

- ⇨ Thank you for noticing this. The following reference is now added after this sentence on page 7 to support the statement that carotid plaque indicates a very high risk of CVD:

  2. Perk J, De Backer G, Gohlke H, et al. European Guidelines on cardiovascular disease prevention in clinical practice (version 2012). The Fifth Joint Task Force of the European Society of Cardiology and Other Societies on Cardiovascular Disease Prevention in clinical Practice (constituted by representatives of nine societies and by invited experts). Developed with the special contribution of the European Association for Cardiovascular Prevention & Rehabilitation (EACPR). Eur Heart J 2012; 33: 1635-701.

For clarity, it would be helpful if the authors specify how carotid plaque was defined in your study, as definitions of plaque vary considerably in the carotid plaque literature.

- ⇨ Thank you for this relevant suggestion. Carotid plaque was defined according to Mannheim consensus. To inform the reader about this, the following sentence and citations are added on page 7, first paragraph:

Carotid plaque was defined according to the Mannheim consensus (19, 21).

19. Nyman E, Grönlund C, Vanoli D, Liv P, Norberg M, Bengtsson A, et al. Reduced progression of carotid intima media thickness by personalised pictorial presentation of subclinical atherosclerosis in VIPVIZA-A randomised controlled trial. Clin Physiol Funct Imaging. 2023;43(4):232-41.

21. Touboul PJ, Hennerici MG, Meairs S, et al. Mannheim carotid intima-media thickness consensus (2004-2006). An update on behalf of the Advisory Board of the 3rd and 4th Watching the Risk Symposium, 13 th and 15th European Stroke Conferences, Mannheim, Germany, 2004, and Brussels, Belgium, 2006. Cerbrovasc Dis 2007; 23: 75-80.

The ATC abbreviation is need to be written in full the first time.

- ⇨ Thank you for recognising this. The ATC abbreviation is now written in full at first mention and the following sentence, on page 8 in the "Inclusion criteria" paragraph, is revised from:

Moreover, only those who received the BMQ questionnaire and with complete answers on at least one subscale, i.e., answering all four questions on the subscales overuse, harm and benefit respectively, were included in the present study on beliefs about medicines and initiation of cardiovascular preventive medications defined as antihypertensive (ATC C03A, C07-09) and lipid-lowering drugs (ATC C10).

To:

Moreover, only those who received the BMQ questionnaire and with complete answers on at least one subscale, i.e., answering all four questions on the subscales overuse, harm and benefit respectively, were included in the present study on beliefs about medicines and initiation of cardiovascular preventive medications defined as antihypertensive (Anatomical Therapeutic Chemical (ATC) C03A, C07-09) and lipid-lowering drugs (ATC C10).

Statistical analysis
The authors need to add the test(s) used for presenting baseline characteristics.

- ⇨ Due to the study design, i.e. a RCT, baseline characteristics should not differ between intervention and control group as the participants have been randomized to participate in either group. The following publication, cited as number 7 in the manuscript, which describe the VIPVIZA intervention effect after one year, does not include p-values either:

  7. Näslund U, Ng N, Lundgren A, Fhärm E, Grönlund C, Johansson H, et al. Visualization of asymptomatic atherosclerotic disease for optimum cardiovascular prevention (VIPVIZA): a pragmatic, open-label, randomised controlled trial. Lancet. 2019;393(10167):133-42.

Accordingly, no p-values will be presented as no statistical tests are used to investigate if there is a difference in baseline characteristics between the group. The first sentence in the "Statistical analyses" section on page 10 is changed from:

Results are presented as frequencies (percentages), mean values with standard deviation (SD) and median with interquartile range (IQR) when suitable.

To:

Baseline characteristics and results are presented as frequencies (percentages), mean values with standard deviation (SD) and median with interquartile range (IQR) when suitable.

Did the authors assess the proportional hazards assumption of the Cox regression model, for example by using log–log plots and Schoenfeld residuals?

- ⇨ Thank you for bringing up this relevant question. Yes, we assessed the proportional hazards assumption of the Cox regression model by using Schoenfeld residuals. To inform the reader about this, the following sentence is added into the last paragraph in the "Statistical analyses" section on page 11:

Moreover, the proportional hazards assumption of the Cox-regression model was assessed by using Schoenfeld residuals.

In the Cox regression analyses, adjustment was made for sex. Please provide the rationale for this choice.

- ⇨ Thank you for highlighting this. To motivate the choice of adjustment in the statistical analyses, the following sentence and references are added as the second sentence in the second paragraph under the title "Statistical analyses" (page 11):

Adjustment was made for sex as it has been found that adherence differs between men and women (25- 27).

Results
The statement “data not shown” regarding the balance between control and intervention groups makes it difficult for readers to assess comparability. The authors are recommended to provide these data in the supplementary material instead.

- ⇨ This is an important aspect, and we agree that the result from these analyses should be provided so the reader can assess the comparability as you state. Accordingly, this result is presented in “Supplementary appendix 4” and the last sentence in the first paragraph in the “Results” section on page 11 is rewritten from:

No significant difference was found between the control and intervention group regarding their beliefs about medicines being overused, harmful or beneficial (data not shown).

To:

No significant difference was found between the control and intervention group regarding their beliefs about medicines being overused, harmful or beneficial (results shown in supplementary appendix 4).

Since initiation of cardiovascular preventive medication is likely to be related to the presence or absence of carotid plaque, this could represent an interesting sub-analysis, similar to the sex-stratified analysis in Table 2 and 3. Have the authors considered conducting such sub-analyses?

- ⇨ This is a really interesting question; however it is not possible to conduct such sub-analysis due to the study design, i.e. only the participants in the intervention group and their physicians got information about presence or absence of plaque but the participants in the control group and their physicians did not. Moreover, no ultrasound results were available to the healthcare professionals, only to the researchers. Nor were any raw data from the ultrasound available to the participants in the intervention group and their physicians as the information provided to the intervention group only comprised a simplified picture of the participants carotid arteries and a color-coded message based on presence or absence of plaque. To clarify for the reader, the third sentence on page 7 under the “Intervention and control” section is revised from:

The pictorial information was given to both the individual and the general practitioner at the health care centre.

To:

In the intervention group, the pictorial information was given to both the individual and the general practitioner at the health care centre.

Moreover, the following sentence on page 7, first paragraph, line 19-20, is revised from:

The control group performed the ultrasound carotid examination, but the patients and their physicians did not receive any information about the results.

To:

This information was not given to the participants in the control group, i.e. the control group performed the ultrasound carotid examination, but the patients and their physicians did not receive any information about the results.

Discussion
The authors need to add references on their statement: “Cardiovascular preventive medications are effectives and proven but still underused” (page 12)

⇨ Thank you for noticing this. The following four references are added to support the statement, i.e. the first sentence on page 14:

3. Visseren FLJ, Mach F, Smulders YM, Carballo D, Koskinas KC, Bäck M, et al. 2021 ESC Guidelines on cardiovascular disease prevention in clinical practice. Eur J Prev Cardiol. 2022;29(1):5-115.

4. Kotseva K, De Bacquer D, De Backer G, Rydén L, Jennings C, Gyberg V, et al. Lifestyle and risk factor management in people at high risk of cardiovascular disease. A report from the European Society of Cardiology European Action on Secondary and Primary Prevention by Intervention to Reduce Events (EUROASPIRE) IV cross-sectional survey in 14 European regions. Eur J Prev Cardiol. 2016;23(18):2007-18.

5. Bradley CK, Wang TY, Li S, Robinson JG, Roger VL, Goldberg AC, et al. Patient-Reported Reasons for Declining or Discontinuing Statin Therapy: Insights From the PALM Registry. J Am Heart Assoc. 2019;8(7): e011765.

6. März W, Dippel FW, Theobald K, Gorcyca K, Iorga S, Ansell D. Utilization of lipid-modifying therapy and low-density lipoprotein cholesterol goal attainment in patients at high and very-high cardiovascular risk: Real-world evidence from Germany. Atherosclerosis. 2018;268:99-107.

The structure of paragraph 2 is not fully consistent with academic argumentation. I suggest revising it using a clearer argumentative framework, for example by applying Toulmin's model of argumentation (page 12, lines 30-36).

- ⇨ Thank you for this advice. We agree that the text in the second paragraph in the Discussion can be clarified regarding the argumentation and how it relates to the previous discussion. To clarify, the following sentence is added at the end of the second paragraph on page 14 and we hope that Reviewer agree that this improves the context and the academic argumentation:

This further supports that also the multi-factorial VIPVIZA intervention can affect beliefs about medicines and accordingly the willingness to initiate cardiovascular preventive medications.

The authors states that guidelines recommend initiation of lipid-lowering therapy when carotid plaque is present. A reference is required to support this claim.

- ⇨ Thank you for noticing this. The following reference is now added to support this claim on page 14.

2. Perk J, De Backer G, Gohlke H, et al. European Guidelines on cardiovascular disease prevention in clinical practice (version 2012). The Fifth Joint Task Force of the European Society of Cardiology and Other Societies on Cardiovascular Disease Prevention in clinical Practice (constituted by representatives of nine societies and by invited experts). Developed with the special contribution of the European Association for Cardiovascular Prevention & Rehabilitation (EACPR). Eur Heart J 2012; 33: 1635-701.

Tables
Fine. Perhaps add whether the HR is unadjusted or adjusted.

- ⇨ Thank you for your suggestion. To further clarify, the first sentence in the table 2 caption is rewritten from:

Effect on time to initiation of antihypertensive treatment in people with perception of overuse, harm and benefit according to the BMQ subscales and sex are presented as HR with 95% CI based on Cox regression model.

To:

Effect on time to initiation of antihypertensive treatment in people with perception of overuse, harm and benefit according to the BMQ subscales and sex are presented as adjusted HR with 95% CI based on Cox regression model.

And the first sentence in the table 3 caption is rewritten from:

Effect on time to initiation of lipid-lowering treatment in people with perception of overuse, harm and benefit according to the BMQ subscales and sex are presented as HR with 95% CI based on Cox regression model.

To:
Effect on time to initiation of lipid-lowering treatment in people with perception of overuse, harm and benefit according to the BMQ subscales and sex are presented as adjusted HR with 95% CI based on Cox regression model

Reviewer: 1
If you have selected 'Yes' above, please provide details of any competing interests.: Not applicable

Reviewer: 2
If you have selected 'Yes' above, please provide details of any competing interests.: Not applicable

Reviewer: 3
If you have selected 'Yes' above, please provide details of any competing interests.: Not applicable

VERSION 2 - REVIEW

**Reviewer**	**2**
**Name**	**Ho, Chau**
**Affiliation**	**Curtin University Bentley Campus**
**Date**	**23-Sep-2025**
**COI**	

Thank you for revising the manuscript and addressing the reviewers' comments. I have reviewed the updated submission and provide the following comments for your consideration:

Concern Regarding the Use of Cox Regression Model: I remain concerned about the appropriateness of using a Cox regression model for the study's main outcome, which appears to be defined as a continuous variable—time to initiation of antihypertensive or lipid-lowering drug treatment (measured in days). The Cox proportional hazards model is typically designed for time-to-event outcomes with censoring, where the event is binary. A continuous outcome, as currently described, is not suitable for Cox regression. Could the authors clarify whether the outcome is the binary event of initiating antihypertensive or lipid-lowering drug treatment (yes/no) or the continuous measure of time to initiation (in days)?

Covariate Selection for Cox Model: please provide a rationale explaining why adjustment for sex alone (rather than adding other characteristics such as CVD risk score, blood pressure levels or serum cholesterol level..) adequately accounts for potential confounding factors.

Clarity of Tables 2 and 3: I find the presentation of Tables 2 and 3 difficult to interpret. It is unclear what the number of events represents in these tables. is it the number of participants initiated the treatment? I'm not quite understand the results of these table. For example in the sentence: "Participants included in the VIPVIZA control group, and with higher scores on negative beliefs about medicines being overused compared to those with lower scores had significantly longer time to initiation of antihypertensive treatment (HR 0.91; 95% CI 0.84–0.996)." Please elaborate on how this hazard ratio (HR) 0.91 is interpreted as "..those with lower scores had significantly longer time to initiation of antihypertensive treatment". Can it be interpreted as those with lower scores were less likely to initiate antihypertensive treatment? Again, need to confirm what is the main outcome of the survival model.

Thank you for addressing these points. I look forward to your response and further revisions to strengthen the manuscript.

**Reviewer**	**3**
**Name**	**Dahl, Marie**
**Affiliation**	**Regionshospitalet Viborg, Cardiovascular Research Unit**
**Date**	**27-Oct-2025**
**COI**	

The authors have thoroughly addressed my previous comments, and I would like to congratulate them on this insightful study.

VERSION 2 - AUTHOR RESPONSE

To the Reviewers:
Thank you for the positive feedback and additional comments which enable us to further strengthen the manuscript! We have taken all your comments into consideration and revised the manuscript as follows.

Comments to the Author:

Thank you for revising the manuscript and addressing the reviewers' comments. I have reviewed the updated submission and provide the following comments for your consideration:

Concern Regarding the Use of Cox Regression Model: I remain concerned about the appropriateness of using a Cox regression model for the study's main outcome, which appears to be defined as a continuous variable—time to initiation of antihypertensive or lipid-lowering drug treatment (measured in days). The Cox proportional hazards model is typically designed for time-to-event outcomes with censoring, where the event is binary. A continuous outcome, as currently described, is not suitable for Cox regression. Could the authors clarify whether the outcome is the binary event of initiating antihypertensive or lipid-lowering drug treatment (yes/no) or the continuous measure of time to initiation (in days)?

- ⇨ Thank you for this relevant question. The outcome is the binary event of initiating antihypertensive or lipid-lowering drug treatment dichotomized as yes =1 or no = 0. We agree that this can be clarified for the reader. Accordingly, the first sentence in the following Abstract section on page 2 is revised from:

Measures: *The primary outcome was time to initiation of antihypertensives and lipid-lowering agents, identified within the time frame from inclusion in the VIPVIZA study until the study participants' three- year follow-up visit.*

To:

Measures: *The primary outcome was time to the binary event of initiating antihypertensives or lipid- lowering agents, identified within the time frame from inclusion in the VIPVIZA study until the study participants' three-year follow-up visit.*

Moreover, the following sentence in the "Study outcome" section on page 9-10 is revised from:

Initiation of either antihypertensives or lipid-lowering drug treatment was defined as the first drug dispensation of at least one of the specified ATC-codes, respectively.

To:

Initiation of either antihypertensives or lipid-lowering drug treatment was defined as a binary event (yes or no) and identified as the first drug dispensation of at least one of the specified ATC-codes, respectively.

Finally, the following sentence in the Statistical analyses section, on page 11, is revised from:

Cox-regression analyses, adjusted for sex, were conducted to investigate how the three general beliefs about medicines affect time to initiation of antihypertensive or lipid-lowering drug treatment (treated as a continuous variable), from inclusion in the VIPVIZA-study until the participants' three-year follow-up visit.

To:

Cox-regression analyses, adjusted for sex, were conducted to investigate how the three general beliefs about medicines affect time to initiation of antihypertensive or lipid-lowering drug treatment, from inclusion in the VIPVIZA-study until the participants' three-year follow-up visit.

And the following sentence is added afterwards:

Time to initiation was treated as a continuous variable and initiation of drug treatment was treated as a dichotomous variable.

Covariate Selection for Cox Model: please provide a rationale explaining why adjustment for sex alone (rather than adding other characteristics such as CVD risk score, blood pressure levels or serum cholesterol level..) adequately accounts for potential confounding factors.

⇨ Thank you for this important and relevant suggestion. This refers to the participants overall CVD risk and accordingly the treatment decision in each individual case. I.e. blood pressure levels and serum cholesterol, which are included in the CVD risk score, affects the treatment decision but there might be more risk factors present, such as being sedentary, obese, having diabetes, familial hypercholesterolemia and/or increased IMT and/or plaque detected by carotid ultrasonography. Information about IMT or plaque were not available for the control group. Accordingly, it would not be possible to adjust equally for these factors among intervention and control group, respectively. Moreover, CVD risk factors did not differ between intervention and control group. We therefore decided to only adjust for sex as this factor is considered the most important and relevant factor due to being a well-known confounder as stated and referenced in the manuscript. To inform the Reviewer about the result when SCORE is included in the model, we conducted sensitivity analyses. The results from the analyses are found in the following two tables.

Table 2. Effect on time to initiation of antihypertensive treatment in people with perception of overuse, harm and benefit according to the BMQ subscales, sex and SCORE are presented as adjusted HR with 95% CI based on Cox regression model. Analyses are stratified for control and intervention group respectively.

**Overuse**					**Harm**				**Benefit**			
	**Control** (events = 71)		**Intervention** (events = 58)		**Control** (events = 71)		**Intervention** (events = 60)		**Control** (events = 71)		**Intervention** (events = 60)	
	n	HR (95% CI)	n	HR (95% CI)	n	HR (95% CI)	n	HR (95% CI)	n	HR (95% CI)	n	HR (95% CI)
BMQ[a]	449	0.92 (0.84-1.005)	429	1.07 (0.96-1.18)	444	0.95 (0.87-1.04)	433	1.04 (0.95-1.14)	450	1.02 (0.91-1.14)	433	0.98 (0.86-1.12)
Sex												
Men	173	Ref.	191	Ref.	170	Ref.	190	Ref.	172	Ref.	190	Ref.
Women	276	2.13 (1.18-3.82)	238	1.57 (0.81-3.04)	274	2.16 (1.20-3.89)	243	1.60 (0.84-3.04)	278	2.18 (1.21-3.91)	243	1.58 (0.83-2.99)
SCORE												
Low risk (<1%)	208	Ref.	179	Ref.	206	Ref.	182	Ref.	210	Ref.	183	Ref.
Moderate risk (1-4%)	231	3.13 (1.71-5.73)	244	2.62 (1.27-5.41)	228	3.23 (1.77-5.91)	245	2.57 (1.28-5.16)	230	3.22 (1.76-5.88)	244	2.60 (1.29-5.34)
High risk (5-9%)	9	10.20 (3.02-34.42)	6	7.45 (1.46-38.02)	9	11.44 (3.35-39.09)	6	6.22 (1.26-30.67)	9	10.84 (3.21-36.59)	6	6.33 (1.28-31.36)

[a] Score range 4-20, 20 = strongly believes, on all four questions within each subscale, that drugs are overused, harmful or beneficial. Events = Number of participants initiating antihypertensive treatment in each group. HR = Hazard ratio, CI = Confidence interval, BMQ = Beliefs about medicines questionnaire, SCORE = Systematic Coronary Risk Estimation

Table 3. Effect on time to initiation of lipid-lowering treatment in people with perception of overuse, harm and benefit according to the BMQ subscales, sex and SCORE are presented as adjusted HR with 95% CI based on Cox regression model. Analyses are stratified for control and intervention group respectively.

**Overuse**					**Harm**				**Benefit**			
	**Control** (events = 48)		**Intervention** (events = 132)		**Control** (events = 48)		**Intervention** (events = 131)		**Control** (events = 48)		**Intervention** (events = 131)	
	n	HR (95% CI)	n	HR (95% CI)	n	HR (95% CI)	n	HR (95% CI)	n	HR (95% CI)	n	HR (95% CI)
BMQ[a]	604	0.94 (0.85-1.05)	564	0.97 (0.91-1.04)	600	0.97 (0.87-1.08)	568	1.02 (0.95-1.08)	606	0.94 (0.82-1.08)	570	1.01 (0.93-1.09)
Sex												
Men	247	Ref.	263	Ref.	244	Ref.	261	Ref.	248	Ref.	263	Ref.
Women	357	0.81 (0.38-1.73)	299	1.09 (0.67-1.76)	356	0.95 (0.46-1.99)	307	1.08 (0.67-1.75)	358	0.94 (0.46-1.96)	307	1.02 (0.63-1.65)
SCORE												
Low risk (<1%)	255	Ref.	210	Ref.	254	Ref.	213	Ref.	256	Ref.	214	Ref.
Moderate (1-4%)	334	1.61 (0.70-3.71)	334	1.05 (0.64-1.71)	331	1.84 (0.82-4.13)	335	1.05 (0.65-1.71)	335	2.06 (0.91-4.69)	336	1.01 (0.62-1.65)
High (5-9%)	14	-	18	1.64 (0.62-4.33)	14	-	18	1.66 (0.63-4.38)	14	-	18	1.57 (0.59-4.17)

[a] Score range 4-20. 20 = strongly believes, on all four questions within each subscale, that drugs are overused, harmful or beneficial. Events = Number of participants initiating lipid-lowering treatment in each group. HR = Hazard ratio, CI = Confidence interval, BMQ = Beliefs about medicines questionnaire, SCORE = Systematic Coronary Risk Estimation

To inform the reader about the rationale behind the statistical models, the following sentence and supporting reference, are added in the “Statistical analyses” section on page 11:

No adjustments for CVD risk factors were made as no information about intima media thickness (IMT) or plaque were available for the control group at baseline and because CVD risk factors did not differ between intervention and control group [7].

Moreover, a reference is added and the following sentence in the “Statistical analyses” section on page 11 is revised from:

Stratified analyses were performed for VIPVIZA intervention group and control group, separately.

To:

Stratified analyses were performed for VIPVIZA intervention group and control group, separately, as the intervention has been found to affect drug initiation [10].

Clarity of Tables 2 and 3: I find the presentation of Tables 2 and 3 difficult to interpret. It is unclear what the number of events represents in these tables. is it the number of participants initiated the treatment?

- ⇨ Thank you for letting us know what is difficult to understand with the present tables. It is correct that it is the number of participants who initiated antihypertensives and lipid-lowering drug treatment, stratified for control and intervention group and with complete answers on each BMQ-subscale. To clarify this for the reader the following information is added beneath Table 2:

Events = Number of participants initiating antihypertensive treatment in each group.

And beneath Table 3:

Events = Number of participants initiating lipid-lowering treatment in each group.

I'm not quite understand the results of these table. For example in the sentence: "Participants included in the VIPVIZA control group, and with higher scores on negative beliefs about medicines being overused compared to those with lower scores had significantly longer time to initiation of antihypertensive treatment (HR 0.91; 95% CI 0.84–0.996)." Please elaborate on how this hazard ratio (HR) 0.91 is interpreted as "..those with lower scores had significantly longer time to initiation of antihypertensive treatment". Can it be interpreted as those with lower scores were less likely to initiate antihypertensive treatment? Again, need to confirm what is the main outcome of the survival model.

- ⇨ Thank you for this comment. The initiation of drug treatment is a dichotomous variable, the time to initiation is measured in days and the BMQ subscales are measured as a continuous variable with higher scores indicating more negative beliefs about medicines, in this case stronger beliefs about medicines being overused. Accordingly, the significant result, HR 0.91; 95% CI 0.84-0.996, should be interpreted as follows. For each increase of 1 point on the BMQ subscale, measuring beliefs about medicines being overused, the relative initiation rate (HR) of initiating antihypertensive drug treatment is reduced by 9%. Thus, participants in the control group, who reported more negative beliefs about medicines being overused had a lower rate of initiation of antihypertensive drug treatment (HR 0.91; 95% CI 0,84-0,996). To make the result easier to interpret the following sentence in the "General beliefs about medicines and initiation of antihypertensive drugs" section on page 11 is revised from:

Participants included in the VIPVIZA control group, and with higher scores on negative beliefs about medicines being overused compared to those with lower scores had significantly longer time to initiation of antihypertensive treatment (HR 0.91; 95% CI 0.84-0.996).

To:

Participants included in the VIPVIZA control group, who reported stronger negative beliefs about medicines being overused had a significantly lower rate of initiation of antihypertensive drug treatment (HR 0.91; 95% CI 0.84-0.996) compared to those with lower scores and consequently weaker negative perceptions.

Thank you for addressing these points. I look forward to your response and further revisions to strengthen the manuscript.
